# Making researchers responsible: attributions of responsibility and ambiguous notions of culture in research codes of conduct

**DOI:** 10.1186/s12910-020-00496-0

**Published:** 2020-07-07

**Authors:** Govert Valkenburg, Guus Dix, Joeri Tijdink, Sarah de Rijcke

**Affiliations:** 1 Centre for Science and Technology Studies (CWTS), Faculty of Social and Behavioral Sciences, Leiden University, Leiden, Netherlands; 2 Present Address: Department of Interdisciplinary Studies of Culture, Faculty of Humanities, Norwegian University of Science and Technology, Trondheim, Norway; 3 Department of Medical Humanities, Amsterdam University Medical Center, Location VUmc Amsterdam, Amsterdam, Netherlands; 4 Department of Philosophy, VU University Amsterdam, Amsterdam, Netherlands

**Keywords:** Research integrity, Codes of conduct, Attribution of responsibility, Research culture, Institution versus individual

## Abstract

**Background:**

Research codes of conduct offer guidance to researchers with respect to which values should be realized in research practices, how these values are to be realized, and what the respective responsibilities of the individual and the institution are in this. However, the question of *how* the responsibilities are to be divided between the individual and the institution has hitherto received little attention. We therefore performed an analysis of research codes of conduct to investigate how responsibilities are positioned as individual or institutional, and how the boundary between the two is drawn.

**Method:**

We selected 12 institutional, national and international codes of conduct that apply to medical research in the Netherlands and subjected them to a close-reading content analysis. We first identified the dominant themes and then investigated how responsibility is attributed to individuals and institutions.

**Results:**

We observed that the attribution of responsibility to either the individual or the institution is often not entirely clear, and that the notion of *culture* emerges as a residual category for such attributions. We see this notion of responsible research cultures as important; it is something that mediates between the individual level and the institutional level. However, at the same time it largely lacks substantiation.

**Conclusions:**

While many attributions of individual and institutional responsibility are clear, the exact boundary between the two is often problematic. We suggest two possible avenues for improving codes of conduct: either to clearly attribute responsibilities to individuals or institutions and depend less on the notion of culture, or to make culture a more explicit concern and articulate what it is and how a good culture might be fostered.

## Background

Research codes of conduct provide guidance for doing research [1]. This guidance ranges from generic advice to strict rules that are subject to sanctions and must be obeyed. While researchers seem the obvious primary agent to bear this responsibility, much of it is actually borne by the institutions where they work. Correct reporting, giving proper references in publications, and showing due respect to patients as research subjects (in the case of biomedical research) might arguably seem to be the clear responsibility of individuals, while other aspects might logically seem to be more the concern of institutions: providing infrastructure for data security, offering research integrity training, and establishing ethics review committees. On closer inspection, however, as we will show, such attributions are not always so clear-cut. Given that this is not a trivial division, we are interested in how the codes make individuals and institutions responsible for realizing a specific value, or for ensuring that research is conducted properly.

This leads us to address the following two research questions in this paper. First, *what are the main values reflected in research codes?* Second, *to whom or to what do the codes attribute responsibility for realizing these values: to the individual, to the institution, or to another entity?* We analyzed 14 institutional, national, and international codes of conduct – mainly in the field of biomedicine – to answer these two questions.

This paper will continue by briefly introducing the two conceptual pillars that guide our analysis: 1) the basic values that are important in biomedical (and other) scientific research, as reflected by research codes of conduct; and 2) the idea of attributing responsibility for realizing these values, dividing it between the individual and the institution. The method will then be explained, after which the results will be presented and analyzed. Importantly, as we will demonstrate, the results give cause to look more closely at how notions of “culture” function in codes of conduct, as something located inconveniently between the individual and the institution with respect to attributing responsibility.

The literature on *responsible conduct of research* (RCR) addresses inter alia research codes of conduct. These codes are essentially normative: they express values that ought to be realized in research practices. To gain a clear picture of how responsibilities are assigned to either individual researchers or research institutions, we need to distinguish between different kinds of values, because they may be associated with different preferences for attributing responsibility.

### Values in research practices

The values enshrined in research codes of conduct have emerged over time and serve different beneficial goals. We discern four main classes of values, each relating to one kind of beneficiary:

1. values that contribute to producing the best possible scientific knowledge,
2. values that uphold good scientific practice and responsibilities to colleagues,
3. values that must be realized in order to be a good researcher oneself, and
4. values that embody the responsibility of scientific researchers towards society.

We arrived at these four classes in the first phase of the study, moving back and forth between the literature on research ethics and the empirical material, consisting of research codes of conduct. Thus, while the classification is grounded in the empirical material, it is also derived deductively from the literature to organize that very empirical material. We will use these classes as a means of organizing our investigation, as they may each have a different relationship to the question of who is responsible for realizing them.

The first class of values, relating to truthfulness and more generally to the proper production of scientific knowledge, often includes values such as methodological rigour, transparency [2], and fair peer review [2–4]. Additionally, more abstract values like inquisitiveness [1, 5, 6] and integrity [7] are frequently mentioned in this respect. The literature draws much inspiration from recent cases of fraud, such as the Stapel case [8] and the Schön case [9]. In this literature, fraud is chiefly understood as *falsification, fabrication, and plagiarism* (FFP) [10–12], and considerable emphasis is placed on their avoidance.

The second class of values (values that uphold good scientific practice and responsibilities to colleagues) concerns how researchers relate to their colleagues. A particularly prominent aspect appears to be fairness in the treatment of authorship and attribution of earlier work, that is to say: proper referencing [13]. Good mentorship [4, 14] and the importance of good role models [6, 15] are also identified in this context. These can be seen as ways to operationalize the recognition that the collective nature of science requires individual members to uphold that communal quality [16].

The third class concerns researchers’ responsibility to maintain their own qualities as a researcher. They should make an effort to keep their research skills up to date, so as to preserve the quality of their contribution to the field. This starts with obtaining the proper formal training before entering a field [17]. In a more abstract sense, it is also described as the need to preserve a good “character” [6, 18, 19]. Given that the ability to do good science requires training and practice, and can easily succumb to compromising influences, a good character of this kind is needed to withstand those pressures [20].

The final class of values is connected with the fact that scientific practice bears a wider societal responsibility. Broadly speaking, this means that scientific research must somehow be useful; it must contribute to a fair and inclusive distribution of wellbeing in society; it must be reliable and truthful in some way (even though we already defined this as an obligation to science itself); and in general it must contribute to the realization of societal values. In the specific case of biomedical research, this explicitly includes a responsibility towards patients, whether or not as research subjects. Influential work in this field [19] has resulted in the formulation of four general principles: autonomy, beneficence, non-maleficence, and justice. While the human research subject is an important subset of “society” towards which research has to act responsibly, we do not feel that our research questions require us to further engage with this particular subset. This class of values is largely, but not exclusively, about justifying the privileged position of science as a producer of truth, and indeed a comparatively autonomous one. Only if the members of the scientific community display indisputable integrity will it be possible to maintain this position.

### Attribution of responsibility

The defining quality of a value is that it is (arguably) desirable. In itself, this leaves unspecified *who* is responsible for realizing that value. It may seem self-evident that each individual, professional researcher bears the responsibility for realizing the values of responsible research. However, there is often no obvious reason why realizing any given value should be an individual responsibility. In many cases, it might be argued just as convincingly that an institution is (partly, if not primarily) responsible for realizing that value. Indeed, the RCR literature has widened its focus to include the responsibility of institutions for facilitating the conduct of research with integrity [9, 15, 21].

Our reason for focusing on attributions of responsibility is that they are not at all trivial, and at times are even problematic. For example, it might seem to be common sense that an individual researcher should display an innovative attitude towards novel research problems. However, the current competitive system has been shown to dissuade people from venturing into innovative research [22]. Hence, if we want researchers to be innovative, institutions bear some responsibility with respect to creating the right conditions. Similarly, publication bias – that is, the tendency *not* to publish results of research if the results are “negative” in the sense that originally expected, positive, significant findings are not found [22, 23] – is generally agreed to be a bad thing and researchers should not allow themselves to be influenced by it; yet this requires that reward systems are arranged in such a way that publishing these negative results is equally as attractive as publishing positive results, which currently appears not to be the case. This means that not only research institutions but also journal publishers bear a responsibility in this regard.

If there is insufficient specification of *who* is responsible, all relationships of accountability and responsibility become ambiguous as well. Consider, for example, a situation where transparency has not been exercised, when in fact it should have been. In this situation, from certain perspectives, both the institution and the individual researcher can be seen as responsible for the misconduct, and it depends on the wider picture who should be held responsible for the lack of transparency. The researcher may indeed simply have ignored procedures for openness; yet the institution in turn may have failed to establish and enforce relevant protocols and failed to provide the researcher with assistance in achieving transparency. The researcher and the institution each have particular features making it possible or impossible to bear part of this responsibility. Only if a code of conduct is sufficiently specific about who bears which responsibilities will it be able to avoid the pitfall of nobody being or feeling responsible; or perhaps worse, a situation where all responsibility is uncritically shifted to the individual researcher.

### Research questions

We investigated research codes of conduct from the specific perspective of the kinds of RCR values that they express and the kinds of entities – individual researchers, institutions, or cultures – to which they attribute the responsibility for realizing these values. Hence, our first research question is: *what are the main values reflected in research codes?* Our second research question is: *to whom or to what do the codes attribute responsibility for realizing these values?*

## Method

The scope of our study is defined as the biomedical research conducted in University Medical Centres (UMCs) in the Netherlands. In addition, other public-sector research is bound by the same rules and regulations, which makes the group of UMCs representative of the sector. We selected all the research codes to which any biomedical researcher in these UMCs is subject. From the highest level of organization to the lowest, they are (1) at the global and European level: the Helsinki Declaration (version 2013) of the World Medical Association, the ALLEA code of the joint European Academies, and the code of the European Science Foundation; (2) at the national level: the joint code of inter alia the VSNU (the Association of Universities in the Netherlands) and the NFU (the Netherlands Federation of University Medical Centres) and the code of conduct for medical doctors issued by the KNMG (Royal Dutch Medical Association); and (3) at the institutional level: the codes of all eight UMCs in the Netherlands (see Table 1). At the time of writing, the Academic Medical Center and the VU University Medical Center, both in Amsterdam, were completing their merger into the Amsterdam UMC. During this process they had already established a joint research code.

**Table 1:** Consulted research codes of conduct

Code of conduct	Issuing party/parties
*International*	
Helsinki Declaration	World Medical Association
Good scientific practice in research and scholarship	European Science Foundation
The European Code of Conduct for Research Integrity	ALLEA All European Academies
*National*	
KNMG Gedragsregels voor Artsen (Code of conduct for medical doctors)	KNMG Royal Dutch Medical Association
The Netherlands Code of Conduct for Research Integrity (draft version 2018; not yet definitive)	KNAW Royal Netherlands Academy of Arts and Sciences NFU Netherlands Federation of University Medical Centres NWO Netherlands Organisation for Scientific Research TO2 Federation of Applied Research Organisations VSNU Association of Universities in the Netherlands VH The Netherlands Association of Universities of Applied Sciences
Wet medisch-wetenschappelijk onderzoek met mensen (Medical Research (Human Subjects) Act) *NOT FURTHER CONSIDERED*	The Netherlands
*Institutional*	
Scientific Integrity at the LUMC	Leiden UMC
Research Code of Maastricht UMC+	Maastricht UMC+
Regeling Wetenschappelijk Integriteit (Academic Integrity Regulations) Radboud Universiteit Nijmegen	Radboud Universiteit Nijmegen
AMC-VUmc Research Code	AMC (Amsterdam) VUmc (Amsterdam)
Erasmus MC Research Codes	Erasmus MC
Research Code for Scientific Integrity of UMC Utrecht & Vision Document: Good scientists make good science	UMC Utrecht
Research Code of University Medical Center Groningen	UMC Groningen

It could be argued that we should also have included the Medical Research (Human Subjects) Act (WMO). However, a prima facie interpretation of that text reveals that it is about the absolute boundaries of acceptable research conduct, articulating explicitly the obligations of both researchers and research institutions. It therefore does not contain any interesting reflections on attributing responsibilities, on the relationship between individual and institution, or on how the boundary between their responsibilities is to be implemented.

If a code was available in both English and Dutch, we used the English version. In cases where only a Dutch version was available, we considered translation to be unproblematic: authors of such texts will typically avoid ambiguity, so nothing is likely to be lost in translation.

We imported all the codes into the *qualitative data analysis* package Atlas.ti. In the first round of analysis, we inventoried the basic values expressed by these codes, as introduced above. Labels^1^ were attached manually by the researchers to extracts of the codes, signifying the value to which reference was made. This involved an interpretive step by the researchers: sometimes a value is explicitly mentioned, but sometimes there is just a description that we interpreted as embodying a certain value. This was done by one researcher [GV], and some doubtful cases were discussed with the others.

In a prima facie analysis of these labelled values, complemented with a literature study, four classes of values were identified. The values were therefore arranged into the classes described above: truth, the scientific community, the researcher personally, and society. A list of the value labels grouped into these four classes is given in Table 2. Examples with text extracts will follow in the Results section. The interpretation and labelling of the values is robust vis-à-vis any researcher subjectivity (corresponding to “inter-coder reliability” in other research designs), as it only organizes the research material for later interpretation. No explanatory power is attached to the labels and classes themselves.

**Table 2:** Values and classes of values

**Truth**	
Bias (avoidance of)	
DataIntegrity	
Disinterestedness	
FFP (avoidance of)	
Impartiality	
Independence	
Integrity	
Rigour	
Selection bias (avoidance of)	
Sloppy science (avoidance of)	
Veracity	
**Colleagues**	
Bullying (avoidance of)	
Collectiveness	
Fairness	
Ghost authorship (avoidance of)	
Leadership	
Mentorship	
Openness	
PsychSafety	
Reliability	
Trust	
**Profession**	
Autonomy	
Character	
Expertise	
Negligence (avoidance of)	
Training	
**Society**	
Accountability	
Communalism	
Conflicts of interest (avoidance of)	
Deception (avoidance of)	
Human values	
Outreach	
Relevance	
Respect	
Responsibility	
Safeguarding dignity	
Sensationalism (avoidance of) Transparency	

In a second round of analysis we inventoried the attribution of responsibility in all the selected text extracts; that is to say, whether and how realization of these values is stipulated, specifically with reference to exactly where the responsibility is placed: on the individual, the institution, or elsewhere. This was also done manually. For example, the extract: “Open communication in discussing the work with other scientists, in contributing to public knowledge through publication of the findings, and when communicating with the general public. This openness presupposes the proper storage and availability of data and their accessibility for interested colleagues” (UMCU Research Code, p. 8) in our view represents the value of “transparency”, and does not clearly attribute this responsibility to either the researcher or the institution. In contrast, the extract: “Researchers across the entire career path, from junior to the most senior level, undertake training in ethics and research integrity” (ALLEA, p. 5) in our view expresses an opinion on the value of “training” and clearly attributes the responsibility to the researcher.

We additionally found it necessary to perform a third round of analysis, looking at notions of “culture”. Here we used an automated search and then assessed and analyzed the occurrences manually, as reported in the Results section below.

We did not seek to assign any explanatory power to the level of operation of the codes and therefore treated all the codes as equal, whether their level is global or institutional, although we noticed some seemingly structural differences between institutional and supra-institutional codes. We also did not make a historical analysis but rather aimed to provide a snapshot of the situation at the time of writing. All of the analyzed codes were either in effect at that time or were in a drafting phase and expected to be in effect soon, in more or less the analyzed form.

## Results

In the following subsections, we discuss the values that emerged from our analysis of the research codes, grouped into the four classes discussed above. We also discuss whether they are explicitly or implicitly attributed to individuals or to institutions, or whether they remain unspecified on this point. We first present a prima facie overview of the codes and then discuss the four classes of values. Finally, we look at how responsibilities within these four clusters are attributed.

### Prima facie assessment of research codes of conduct

The codes differ greatly in length and territory. Of the institutional codes, the longest (joint code of AMC and VUmc) is 64 pages in length and the shortest (Radboud UMC) is nine pages in length (no explanatory power is assigned to the number of pages: this remark is only intended to indicate their diversity; it should also be noted that the layout of all the documents was full-text A4 size with few or no graphics). The longer the codes are, the broader the range of issues they cover. For example, the Erasmus MC code (47 pages) is explicitly divided into three parts (academic integrity, intellectual property, and patient data and material). And while the code of UMC Utrecht is relatively short, at 31 pages, much of the territory is covered in the parallel 148-page Vision Document (bilingual). The fact that the codes are very different in terms of their length and the territory they cover makes it hard to compare them with respect to how strict they are, or how much attention they devote to specific issues. Moreover, if something is missing from a code, there is no reason to assume that the institution is indifferent to that issue: it might simply be resolved in a different way, through other means than this particular form of codification.

Compared with these institutional codes, the supra-institutional equivalents are very short; for example, the WMA code has only two pages, and the KNMG code only five, plus a two-page preamble. The main reason for this difference seems to be that the institutional codes contain much more specific implementation: exactly which committees and procedures are in place, what policy aims correspond to the expressed norms and values, and how the codes themselves are to be regularly monitored and revised by the institution and its members. We have no reason to assume that it is particularly “Dutch” to write long codes, and we attach no further significance to the number of pages than these broad-stroke observations.

After a first reading of all the codes, in parallel with the literature study reported above, we iteratively produced the classification of four value categories. In principle, this could result in some values being overlooked because they do not fit any of the four categories. Therefore, we first identified all value statements and only then imposed the categorization and ascertained that all of the value statements were indeed covered by one of the four categories.

### Values

#### Truthfulness

The first class of values consists of those principles that are conducive to producing “true” knowledge. They thus specify how researchers should engage with their objects of research, how knowledge should be presented, how knowledge is corroborated, etc. While we use the single term “truthfulness” here, this is in fact a complex concept: in practice, it involves many constitutive principles and indeed potentially conflicting ones; and in codes it is usually made evident through more specific and substantive principles.

One way in which truthfulness typically manifests in codes is through an emphasis on *rigour*: scientists should do their work properly and according to the current standards of the field. It is not always explicitly defined under this heading, but the KNAW code (p. 8), for example, states: “Scrupulousness means using methods that are justified or seen as the standard within the discipline and exercising the best possible care in designing, executing, reporting and disseminating the research.” Expressions found in institutional codes include meticulousness, attention to detail, proper reporting and referencing, absence of deceit, and the fact that an individual should undergo appropriate training to achieve rigour. Other instances of this endeavour for truthfulness are values such as disinterestedness and impartiality. As the UMCU code (p. 4) states: “Research is to be independent of commissioning or interested parties, ideological or political pressure groups, and economic or financial interests. Any limitation of academic freedom needs to be made clear.” And the MUMC+ code (p. 7, our translation) states: “Academic practitioners will not allow their research activities to be guided by non-scientific or non-scholarly considerations. [ …] They operate in a context of academic freedom and impartiality. Insofar as constraints on this freedom are inevitable, they will be disclosed.”

In a general sense, ideals of what is good may consist of absence and prevention of what is bad, and truthfulness is indeed often presented as the avoidance of untruthfulness. In fact, research codes largely consist of references to avoidance of fraud, defined as *falsification, fabrication, and plagiarism* (FFP), and avoidance of problems such as conflicts of interest, evasion of laws and regulations, and failure to remedy misconduct. For example, in the ALLEA code (chapter 3), FFP is explicitly described as the “traditional definition” of research misconduct; and in the Radboud code, it is a central focus of the appendix that defines violations of scientific integrity. It is striking that in this latter code the first seven of the nine pages are devoted to the formal arrangements and procedures for dealing with misconduct, while the last two pages concern the definition of misconduct, with five of the eight points relating to FFP or similar violations of the truth.

#### Colleagues, practice and community

The second class is collegiality: the obligations and entitlements that derive from membership of a research community, notably towards colleagues, the institution and the broader academic community. All the codes express in some way that research is a collective affair, and that the individual has a responsibility towards this collective nature. One value subsumed under collegiality is fairness about authorship. Most of the codes contain some reference to the duty to acknowledge other people’s work. For example, in the Radboud code, two points in the appendix concern authorship issues. Interestingly, the UMCU code (p. 19, our translation) also specifies what is *not* a sufficient ground for authorship: “For the sake of clarity, it is important to state that obtaining a grant, collecting data, and/or generally managing a research group is NOT sufficient to warrant authorship.” Thus, this institution claims at least some of the responsibility in setting the standards for authorship. There is also a generally recognized duty to take responsibility for the conduct of others. Most of the codes make reference to fallback arrangements, such as confidentiality officers and protection for whistleblowers. For example, the UMCU code (p. 8), the AMC-VUmc code (p. 57), and the Radboud code (p. 1) all mention explicit arrangements for whistleblowers. The supra-institutional KNAW code (p. 14) explicitly stipulates that establishing such arrangements is the responsibility of institutions. While the above quotations provide ample reason to trust that institutions do indeed accept this responsibility, it is interesting to see that it was evidently deemed worthwhile to make this explicit.

#### Professionalism

The codes also express a responsibility for what it takes to become or remain a good researcher, possessing the skills needed to fulfil the values that constitute good science. We label this class *professionalism*. Given the responsibilities borne by researchers, it is not an entirely voluntary matter for them to work on their professionalism. In fact, it is the very condition for their acceptance to do this kind of work. While the aim of codes of conduct is to specify good research, they are usually not explicitly worded in terms of what a good researcher is. One exception that refers to a character trait is the LUMC code: under the heading “Profile of an honourable researcher” it explains many of the more operational values, such as being respectful, meticulous, impartial, and responsible. The codes also describe some more specific personal qualities, an important one being the need to engage in training. The ALLEA code (section 2.2) explicitly states that researchers should undertake training across their entire career path. The KNAW code (section 5.2) stipulates that institutions should ensure that education is provided, and particularly education in research integrity.

#### Values that benefit society

The final entity towards which medical research is said to bear a certain responsibility is society at large. This relates to the contribution made by science to the benefit of society. The values in this class explicitly address society as an actor or actor category that has a stake in the value being realized, while the first three classes of values contribute more indirectly to societal benefit.

*Accountability* and *transparency* are important guises in which societal responsibility is observed. Strictly speaking, accountability is quite different from transparency: the former involves placing oneself in a position where one can be held responsible, while the latter is about providing information on the process underlying specific knowledge outcomes. However, the connection between them is easy to see, and indeed the two are often aligned in their operationalization in codes. They both entail clear and complete communication (e.g. KNAW, p. 8; AMC-VUmc, p. 15; MUMC+, p. 7), make it possible for researchers to subject one another’s work to (constructive) discussion (AMC-VUmc, p. 5; UMCG, p. 7), require facilities for archiving material to make it available for cross-checking (UMCU, p. 16; UMCG, p. 19), and in a general sense enable researchers to explain and justify how research outcomes have been obtained.

Another value to nurture in relation to society is that of *relevance*, which in biomedical research primarily means clinical relevance. It appears in many codes, although there is rarely any clear specification of what exactly makes something relevant or not. One interesting remark is found in the LUMC code (p. 28), which suggests that involving patient organizations could help with determining research questions that are relevant. Thus, even if relevance itself is not directly expressed, its procedural definition provides guidance. The connection with society as the recipient of relevant knowledge is stipulated as a criterion here.

### Attributions of responsibility

In the previous section, we inventoried the values that we found to be central to codes, and categorized them into the four classes: values serving truth, values serving colleagues, values upholding the profession, and values benefiting society. Within each of these four classes, we now further explore how the values are presented as responsibilities of individuals or institutions.

#### Attributing responsibility to individuals and institutions

In truth-related values, FFP is the main counterposition that the codes seek to combat. FFP is typically something an individual commits. Interestingly, nowhere in the codes did we see any indication that an institution can be found guilty of such conduct, which suggests that it is indeed always ultimately regarded as an individual responsibility. Similarly, the observed expressions of rigour as the main path to truthfulness – such as meticulousness, attention to detail, proper reporting and referencing, absence of deceit – are hard to see as anything other than individual qualities. However, particularly at the international level, the ALLEA code (section 2.1) specifies that institutions have a responsibility in creating the infrastructural conditions to actually deliver rigour, which includes provision of training. Moreover, establishing clear fallback procedures, such as an ombudsman and whistleblower protection if misconduct has occurred, is generally regarded as an institutional responsibility. Thus, even though truthfulness is primarily an individual duty, a broader view of the context of truthfulness shows that this is not strictly the case, and it can also be an institutional duty.

Regarding the research practice in which the community of colleagues operates, mentorship was emphasized as an important value to realize. While the practical action of mentoring is performed by an individual, many of the codes make clear that mentorship can only take place if the institutional conditions are conducive to it. Mentorship is described in several of the codes as an important instrument in ensuring research integrity. For example, the AMC-VUmc code includes “Good mentorship” as a chapter of its own, providing a host of specific, operational guidelines for what good mentors (including but not limited to PhD supervisors) should do on a day-to-day basis. Similarly, it is also a separate chapter – albeit somewhat shorter – in the UMCG and MUMC+ codes. Mentorship is something that is “done” by an individual mentor, but at the same time it is “enabled” or “stimulated” by the institution, for example by setting standards for mentorship arrangements and providing training to mentors. Two other important duties towards the community, namely fairness to others (chiefly related to authorship) and fairness about others (chiefly related to reporting misconduct), are typically framed as individual duties, although here again it is often recognized that the institutional conditions need to be conducive.

Duties towards the profession are mostly expressed as the need to maintain one’s ability to meet the standards of the field. This is primarily achieved through training, both before entering the professional field and after becoming a member. This is stipulated widely, both by higher-level codes (ALLEA, chapter 2; KNAW, section 5.3) and by institutional codes (UMCU, p. 14). Especially the codes at the supra-institutional level stipulate that there is an important duty to facilitate training at the institutional level. But ultimately, of course, it is the individual researcher who has to take the relevant refresher courses, and we did not find any reference to means of coercion available to institutions.

The duties of research towards society are principally defined as beneficence, accountability, and transparency. For example, the LUMC code (p. 18) explicitly identifies relevance as something that individual researchers should ensure in their research. Another instance is found in the UMCU Vision Document (p. 60), where it is similarly framed as a duty for the individual researcher to have a realistic view of research outcomes and not to promise too much. An interesting point in this respect is that the LUMC (p. 28) recommends that patient organizations should be involved in research. Thus, the institution takes responsibility for setting a standard here, but it appears that the researcher is still ultimately responsible for actually organizing this engagement. The Radboud code (p. 8) states that academic misconduct damages society and the image of research in society, and that the researcher’s employer has primary responsibility for preventing such misconduct. The preamble to the VSNU code (p. 3) states that doing scientific research in service of society is a matter for individual researchers, but it is also important for “their managers and [the board members of] the institutions where they work.”

We did not find any direct or clear-cut relationship between the four beneficiaries and who is supposed to take responsibility for realizing these values, but we have nevertheless been able to reach the following conclusions:

- All the classes contain at least some reference to both individual responsibilities and institutional responsibilities.
- In the two classes of truth-related and society-related values, attributions to the individual are more prevalent than attributions to institutions.
- Obligations towards the profession and obligations towards the research community are more evenly balanced between individual and institutional responsibilities.
- Codes at the supra-institutional and international levels (e.g. KNAW, section 5.6; ALLEA, chapter 2) are generally much more articulate and explicit about the institutions’ responsibilities than the codes of institutions themselves are.

With respect to the truth-related values, this tendency towards individual responsibility might not seem surprising, as these values are much more connected with the practical work of scientific research. However, this tendency is less self-evident with respect to society-related matters. After all, many (but not all) of the science-society relationships are structured at the institutional level and not at the level of the individual researcher.

The attribution to institutions in the higher-level codes is comparatively well operationalized, and they specify such concrete measures as establishing ethics and integrity committees and facilities on institutional websites for information that needs to be published. To some extent this is unsurprising, as these are actually intended to inform individuals rather than institutions; this is simply a natural consequence of different levels of organization entailing different perspectives. Nonetheless, this could be taken to indicate a blind spot in institutional codes. It also appears that the codes are not entirely homogeneous across the board with respect to balancing individual and institutional duties.

#### Attributing responsibility to culture

In the foregoing we have discussed attributions of responsibility to either individuals or institutions and observed that the distinction between the two is not always clear. In addition, during the inductive content analysis we identified a third category, which is specifically situated between the individual and the institution: the notion of *culture*. It also goes by other names – including climate, atmosphere, and environment – and sometimes even remains implicit. It is apparent in references such as:

“As a medical and biomedical scientific research institute, the LUMC is more than the sum of the individual LUMC researchers. Mutual trust between LUMC researchers and research groups is the foundation on which joint research projects are set up, synergy is generated and bigger steps towards knowledge increase can be taken. In this kind of **environment**, medical and biomedical science and education can flourish, and the LUMC can stand out among its competitors in the Netherlands and abroad.” (LUMC, p. 10; our emphasis).

And:

“The UMCG aims to establish a safe **climate** for reporting and acknowledging violations of academic integrity.” (UMCG, p. 27; our emphasis).

We decided to search the documents for the terms “culture”, “climate”, “atmosphere”, and “environment” and their Dutch equivalents (*cultuur, klimaat, sfeer*, and *omgeving*). It should be noted that lexically these terms refer to different things: culture is not the same as environment, for example. However, in our interpretation they do, in fact, refer to the same category when used in the codes. We have counted the (hypothetical) claim that “researchers should jointly establish a culture of integrity” as equivalent to the claim that “researchers should jointly establish an environment of integrity” and so on. This search yielded 92 quotations that referred to any of these terms (henceforth clustered under “culture”). We assessed these occurrences manually.

In the codes, culture is often linked to the existence of well-established values and procedures. For example, the codes sometimes mention the need to establish specific modes of operation, such as organized peer cooperation and evaluation, and placing integrity on the agenda of personal assessment cycles (UMCU code, p. 5). Several codes also refer to the importance of whistleblowing procedures and to ethics review committees and integrity committees (e.g. Radboud UMC, article 4). Combining values with procedures, the KNAW code (article 5) describes the habit of openly discussing dilemmas and the existence of data management facilities as essential elements of a good research culture. In this procedural sense, culture and its equivalents are sometimes linked to specific values, such as transparency, independence, and trust (e.g. AMC-VUmc, p. 54; LUMC, p. 10). Most of the codes explicitly state that openness, psychological safety and safety of reporting contribute to such values (e.g. KNAW, article 5; UMCG, p. 27). It should be noted that this procedural approach to culture shows considerable overlap with what we earlier described as attribution to institutions. However, as the codes *themselves* refer to culture or its equivalents, we regard this as significant: it is apparent that the codes are pursuing something beyond the procedures and hence beyond the institutions themselves.

In addition, culture is sometimes operationalized as the selection of senior (and other) researchers with the right leadership and mentorship characteristics to convey the importance of these values and procedures to a future generation of researchers. The KNAW code (section 5.3), for instance, explains the notion of culture as establishing clear instructions and, insofar as persons are concerned, selecting “the right senior researchers” (section 5.3, point 9). The code does not define here what “right” specifically means. Several codes make reference to the effect that “senior researchers are the bearers of culture” in terms of mentorship, leadership, and the proper design of research projects (e.g. ALLEA, p. 5; UMCU Vision Document, p. 87; AMC-VUmc, p. 56). In a similar vein, the LUMC code (p. 10) devotes nearly a paragraph to creating an “honourable scientific environment” and sees this environment as closely bound up with the need to have the right kind of people in leadership positions. Mentorship is equally recognized as a vital mechanism for perpetuating any kind of “culture”. Collectively held norms are transferred through mentors, insofar as they are not written down on paper. One code regards senior researchers as the “conveyors of culture” (UMCU, p. 87). In the other codes, mentorship can be interpreted along the same lines, even though they do not always make explicit reference to culture. Much like the procedural approach to culture discussed above, this mentorship-based approach seems to suggest something that exists beyond the individual and his or her actions.

In a few cases, culture is mentioned as something that is potentially bad. In one code at the supra-institutional level, for example, culture is depicted as the bearer of sloppy habits and worse. This code then states that particular attention should be given if “the conduct has occurred on more than an occasional basis, for example if the conduct forms part of the research culture in which the researcher works” (KNAW, p. 16). Another institutional code refers to today’s “culture of publish or perish” as a potential cause for researchers being seduced into misconduct (LUMC, p. 18). However, the same code also refers to individuals’ responsibilities, saying that the institution “expects its researchers to take the following steps to avoid scientific fraud, misconduct and plagiarism.” In an interesting parallel move, the UMCU Vision Document (p. 14) stipulates that a researcher with integrity will never invoke environmental factors, including perceived pressure, to justify misconduct: “A researcher with integrity will never shift the blame to others, nor appeal to environmental factors such as performance pressure, in order to justify their actions.” Interestingly, this is not so much an attribution of responsibility to something beyond individuals and institutions, but rather is referring to the existence of something beyond these entities, which has to be countered by individual and institutional responsibilities.

All in all, there are frequent references to culture and its equivalents in the codes of conduct we analyzed. This is an indication that a responsible research culture is an important concern to the writers of the codes and to the wider community of biomedical scientists from which these writers were selected or who were consulted in the writing process. As a rough definition, culture is something that appears to transcend the individual researcher but is too informal for the institution to set in stone with rules and regulations. Or conversely: it can be achieved in part by rules and regulations, but then it has to bring something “bigger” into existence. Despite the marked importance of positing general ideas about culture in research codes, most of the references to culture do not contain any substantial specification of how it is thought to operate. That is, there seems to be no coherent idea of how it emerges and “does” such things as coercing, enabling, or constraining. On a more interventionist note, these codes do not provide detailed guidelines for how to “do” a responsible research culture: their discussion of culture includes no hints about who should do what to foster responsible research practices. As such, it is unclear which specific good mentorship practices should be attributed to culture and which to individuals, or even to institutions. Moreover, in the case of bad research cultures, the codes slide from acknowledging potential cultural influence to emphasizing (mostly) individual responsibilities. If we were to push this to a conclusion, it could in fact mean that all references to culture are actually concealed individual attributions.

## Discussion

### Summary of findings

The main findings of our analysis of research codes of conduct are that (1) the attribution of responsibility for good research practices to individual and institution is sometimes ambiguous, and (2) evoking culture as a category in-between the individual and institutional level has done little as yet to ameliorate this ambiguity. Where we find unambiguous attributions, we see that supra-institutional codes have a stronger tendency to attribute responsibilities to institutions than the codes of the institutions themselves. In the two classes of truth-related and society-related values, attributions to the individual are more prevalent than attributions to institutions. Obligations towards the profession and obligations towards the research community are more evenly balanced between individual and institutional responsibilities.

### Implications

In view of the fact that most of the responsibility is ultimately placed on the shoulders of individuals, we must expect that whenever attribution of responsibility becomes ambiguous – through evocations of culture or otherwise – it will de facto be attributed to individual researchers. This sketches an altogether worrisome picture of responsibility being obscurely imposed on individuals.

Our research points at two possible ways out of this problematic situation. One strategy for improving research codes of conduct is to be more clear-cut about the balance between individual and institutional responsibilities. This *strategy of disambiguation* pertains especially to institutional codes, as the supra-institutional codes already offer a better balance in this respect. If clarification is successful, the references to culture can be rephrased as either individual or institutional responsibilities.

However, the very frequency of references to culture suggests that there is a need to refer to a factor that somehow falls in-between the levels of individual and institution. Even though culture is a difficult concept to grasp and operationalize, certainly for the purpose of setting policy, this widespread use also reflects an intuitive recognition of its importance for how practices ultimately operate. It is implicitly recognized as a source of obduracy: if people are part of a sloppy research culture, then training, arrangements, and rules about the responsibility of the individual researcher are insufficient.

A second strategy – and the one that we would advocate – is the *strategy of articulation*: that is, to explicitly give expression to the notion of culture, which is currently not done in the codes. We could start from a definition of culture such as the one offered by Geertz [24]: “a historically transmitted pattern of meanings embodied in symbols, a system of inherited conceptions expressed in symbolic form by means of which men [sic] communicate, perpetuate, and develop their knowledge about and attitudes towards life.” Carrying this notion to its conclusion for research codes of conduct would require an explicit connection to the symbols, conceptions, and knowledge structures of respective research practices. Starting out from Geertz’s general definition, we could further differentiate between a range of epistemic cultures [25]: different disciplines and different professional communities working within these disciplines have different cultures of producing knowledge. To engage in a particular scientific practice presupposes a long process of becoming acquainted with scientific ideas and assumptions as embedded in specific scientific skills, instruments, methods, and patterns of cooperation. Building on grid-group cultural theory [26, 27], it would be possible to differentiate between the relations with peers (group) and the formalized rules and regulations (grid) that are characteristic of an epistemic culture. Or, alternatively, we could differentiate between the normative, regulative, and cognitive elements within a particular epistemic culture [28].

In addition, there is a certain reflexiveness to talking about culture in insufficiently substantiated ways: it contributes to precisely *the opposite* of a good working culture. If junior researchers are told by their mentors that a good culture is important, but they are not given a clear explanation of what this culture is, the worst case is that they will adopt the same habit of improper explanation and will sweep their responsibilities under a carpet of fancy yet vague words. Conversely, explaining what culture is and how it is operationalized is, in itself, an example of a good culture.

### Limitations

We used four classes of values that were established by moving iteratively between the empirical material and the literature. The upside is that our analysis speaks to both. However, a categorization entirely based on the empirical material (in line with the basic ideas of *grounded theory*) or a categorization entirely based on the literature might have thrown light on different connections or mechanisms. Notably, we have not found a clear explanation, or even an educated guess, as to when writers of codes need recourse to a notion such as culture, using it in comparatively vague ways.

The manual method of labelling text is time-consuming and may miss out on some insights that would be provided by quantitative and automated analyses. At the same time, however, we feel that this method does more justice to the actual meaning of the text as intended by the authors. We also feel that an automated analysis would not be able to deal properly with ambiguities in the text, which emerge here especially in relation to notions of culture.

The codes were taken from the Dutch context, which means that it may not be possible to generalize our findings to other countries. However, the codes clearly show a high degree of diversity in their exact form and formulations, and we did not find any radical deviations from the international context. Thus, we have no reason to expect that the situation is fundamentally different in other countries, at least to the extent that institutions in those other countries subscribe to similar international standards; however, this has not been researched. Moreover, the codes relate to biomedical research contexts, and codes of general universities were only used insofar as they are explicitly applied in the context of a university medical centre (specifically, Radboud UMC and Radboud University have a joint code). Generalization of our results to universities and other research institutions might require additional steps that were left out of the current analysis, but we see no reason why UMCs cannot be taken as a valid strategic research site.

## Conclusions

Research codes of conduct are normative in the sense that they convey the epistemic and moral rules with which researchers and research institutions ought to comply. Under the heading of “values” we discussed four classes of such rules: truthfulness, collegiality, professionalism, and benefit for society. In addition to this basic source of normativity, the codes are also normative in a more specific sense of attributing responsibility to particular actors. Our analysis shows that this second source of normativity is mobilized to enforce different distributions of responsibility: to the individual, to the institution, and to culture.

Insofar as explicit attribution is found, it is more frequently to the individual than to the institution. This should perhaps not come as a surprise: by their very nature, codes are framed as guidelines that primarily address the researcher; research is also intuitively regarded as something that is “done” by human beings; and finally, it is easier to impose sanctions on individuals than on institutions, at least for single instances of misconduct. This is not to say that no responsibility is attributed to institutions. It is broadly acknowledged that they must facilitate researchers in actually being able to work with integrity and to conduct responsible research. In particular, offering training and providing fallback facilities, such as confidentiality officers and ethics review committees, are widely mentioned as responsibilities of institutions. Additionally, more standard infrastructural arrangements, such as education on responsible research, proper information management systems for research data, and guidelines for the checks and balances on any research project, are generally regarded as facilities that an institution should provide. This institutional responsibility is emphasized more in supra-institutional codes than in institutional ones.

It has become clear that research codes of conduct do not always clearly attribute responsibilities to either the institution or the individual. Sometimes they move beyond individual and institutional responsibility. We observed that this area is covered by referring to the notion of culture. We see culture as important in codes of conduct for matters that cannot be reduced to the level of the individual researcher but are hard to formalize into institutional rules or procedures. We also observed that such references are often insufficiently substantiated. We did not find any good explanations of what culture is or how it operates. Up to the present, culture has not been rendered a distinct and well-developed category in addition to the categories of individual and institution.

Codes of conduct are not only interesting objects of analysis but also interesting instruments for intervening in research practices. More articulate attribution of responsibility will help to prevent interventions being made at the wrong level or place. There is little point in remedying a wrong through mentoring if it is actually the consequence of a binding rule; or remedying a wrong at the level of individual action if it is actually the consequence of collective, aggregative action. Taking a reflexive approach to where responsibility should be located is a first step in ensuring that what should be done by an institution does not end up as an individual’s responsibility and vice versa. The current codes fall short of tapping into the potential of interventions that a developed notion of culture would afford. “Building a certain culture” would require being specific about the content of that culture’s meanings, norms, and values. Giving concrete expression to culture is not straightforward and requires specification of how the ideal practice is envisioned, what values are represented and deemed important, what mechanisms should be used in including and excluding its members, and how the existence of the practice is thought to be legitimated. The work of articulating what culture means in a setting of responsible research practices and the codes that befit this would be a first step in the direction of better and more targeted interventions.

## Data Availability

Not applicable; the data used in the study are publicly available from the original authoring institutions.

## Abbreviations

ALLEA: All European Academies
AMC: Academic Medical Center
FFP: Falsification, Fabrication, Plagiarism
KNAW: Royal Netherlands Academy of Arts and Sciences
KNMG: Royal Dutch Medical Association
LUMC: Leiden University Medical Center
MUMC+: Maastricht University Medical Center
NFU: Netherlands Federation of University Medical Centres
RCR: Responsible Conduct of Research
UMC: University Medical Center
UMCG: University Medical Center Groningen
UMCU: University Medical Center Utrecht
VSNU: Association of Universities in the Netherlands
VUmc: VU University Medical Center
WMA: World Medical Association

## Footnotes

1 In qualitative data analysis, these labels are usually referred to as “codes”. However, as this article deals intensively with “research codes” as the unit of analysis, we use “label” here instead to avoid textual ambiguity.
